# The Effect of Cu-Rich Nano-Precipitation on Hydrogen Embrittlement Performance in a Cu-Bearing Ultra-Low Carbon Steel

**DOI:** 10.3390/ma19183971

**Published:** 2026-09-18

**Authors:** Haitao Cui, Mengqi Wang, Yuan Gao, Zhanjie Gao, Haicheng Liang, Jinsong Liu, Weina Zhang

**Affiliations:** 1 School of Materials Science and Engineering, Shenyang Ligong University, Shenyang 110159, China; 2 State Key Laboratory of Digital Steel, Northeastern University, Shenyang 110819, China; 3 Shenyang National Laboratory for Materials Science, Institute of Metal Research, Chinese Academy of Sciences, Shenyang 110016, China

**Keywords:** Cu-rich nano-precipitates, hydrogen embrittlement, microstructure, dislocation, Cu-bearing ultra-low carbon steel

## Abstract

A low-carbon Cu-bearing marine structural steel was fabricated based on the chemical composition of HSLA-100 steel. The microstructural evolution, variation in nanoscale Cu-rich precipitates, and their synergistic effects on the mechanical properties and hydrogen embrittlement (HE) behavior under different tempering durations were systematically investigated. The results showed that the original lath bainite gradually transformed into tempered bainite and equiaxed ferrite with a prolonged tempering time. The Vickers hardness exhibited a typical upward-then-downward trend and reached a peak value of 322 HV at the tempering time of 1 h. The average size of Cu-rich precipitates increased from 7.2 nm to 13.2 nm, while the number density rose rapidly and finally stabilized. The as-rolled sample exhibited the minimum plastic loss after hydrogen charging owing to the hydrogen-trapping effect of high-density tangled dislocations. Short-time tempering (0.5 h) generated fine Cu-rich precipitates with a weak hydrogen-trapping capacity and abundant mobile dislocations, resulting in severe HE deterioration. The optimal HE susceptibility was achieved after 1 h of tempering. Combined with our experimental microstructure-property results and previous published literature, this improvement is inferred to originate from the hydrogen-trapping effect of adequately grown Cu-rich precipitates, together with a substantial decrease in mobile dislocation density. Excessively long tempering (2 h) induced irreversible temper brittleness and grain boundary deterioration, which aggravated the hydrogen-induced plastic degradation. This work clarified the coupled regulation mechanism of the Cu precipitate morphology, dislocation configuration, and temper brittleness on the HE performance of low-carbon marine steel, providing a reliable theoretical basis for the process optimization and anti-hydrogen damage performance improvement of high-strength marine steels.

## 1. Introduction

With the rapid advancement in marine engineering and shipbuilding technology, high-performance structural steels have become indispensable foundational materials for manufacturing deep-sea service equipment, large-scale vessels, and offshore infrastructure [1,2,3]. Advanced hull and offshore steels are required in order to integrate the high strength, excellent low-temperature toughness, and superior weldability to adapt to complex and harsh marine service environments. The HSLA series steels developed in the United States have long been recognized as the international benchmark for high-performance hull structural materials due to their comprehensive mechanical performance and stable service reliability [4,5]. In contrast, conventional high-strength marine steels widely adopted in China mainly relied on traditional Ni–Cr–Mo alloy systems, which frequently suffered from an insufficient strength margin and poor weldability. These inherent limitations restricted the further upgrading and large-scale engineering application of domestic high-end marine steels. Therefore, the development of novel ultra-low-carbon structural steels with a balanced strength, low-temperature toughness, and weldability has become an urgent demand for the high-quality development of modern marine equipment manufacturing [6,7].

Reducing the carbon content is a classical and effective strategy to improve the weldability and low-temperature toughness of high-strength steels, which can effectively mitigate the welding brittleness and enhance structural safety under low-temperature marine conditions [8]. Nevertheless, the ultra-low carbon design inevitably weakened the solid-solution strengthening effect and resulted in a noticeable deterioration in the matrix strength. Copper microalloying provided a feasible solution to resolve this strength–toughness–weldability contradiction. Nanoscale Cu-rich precipitates formed during thermal treatment produced prominent precipitation strengthening, which effectively compensated for the strength loss caused by carbon reduction. Consequently, Cu-modified ultra-low-carbon steels achieved the excellent synergistic optimization of the mechanical performance and welding applicability, demonstrating great potential for advanced marine structural applications.

Despite the superior comprehensive performance of Cu-bearing ultra-low-carbon steels, hydrogen embrittlement (HE) remained a critical threat to their long-term service reliability in humid and hydrogen-rich marine environments. HE is a catastrophic failure behavior in high-strength metallic materials, in which diffusive hydrogen atoms accumulate under external tensile stress, inducing severe plastic deterioration and brittle fracture [9]. Such hydrogen-induced failure significantly shortened the service life of marine steel structures and endangered operational safety. It is generally accepted that HE susceptibility is predominantly governed by the diffusion behavior and local aggregation of hydrogen in the steel matrix [10,11]. To explain the HE mechanism in hydride-free alloys, multiple classical theories have been proposed, including the adsorption-induced dislocation emission (AIDE), hydrogen-enhanced localized plasticity (HELP), hydrogen-enhanced decohesion (HEDE), and hydrogen-enhanced stress-induced vacancies (HESIV) mechanisms [12,13,14,15,16]. Although controversies still existed among these theoretical systems, a universal consensus was reached that suppressing hydrogen diffusion and limiting local hydrogen enrichment could effectively reduce the HE sensitivity of high-strength steels.

Microstructural hydrogen traps played a decisive role in regulating the hydrogen diffusion and accumulation behavior. The hydrogen-trapping capacity was quantitatively evaluated by the hydrogen binding energy, which directly determined the restraint degree on diffusible hydrogen atoms. The perfect iron crystal matrix exhibited an extremely weak hydrogen-binding capacity of approximately 5 kJ/mol, which can be regarded as the baseline of free hydrogen diffusion. In comparison, 30 kJ/mol and 58 kJ/mol are widely adopted threshold values to categorize hydrogen traps. Traps with binding energies below 30 kJ/mol are regarded as low-energy traps, those ranging from 30 kJ/mol to 58 kJ/mol are classified as medium-energy traps, and traps with binding energies above 58 kJ/mol are defined as high-energy traps. Local stress fields formed around lattice defects and secondary-phase particles could interact with hydrogen atoms, adsorb and immobilize diffusive hydrogen, and, eventually, modulate the HE susceptibility of steel materials [17,18,19,20,21].

At present, the mainstream research on hydrogen-trapping modification primarily focuses on carbide precipitates formed by conventional microalloying elements such as Nb, V, and Ti. These carbides can refine grains, induce precipitation strengthening, and suppress the formation of brittle cementite during tempering. Meanwhile, their NaCl-type face-centered cubic crystal structures enabled effective hydrogen trapping at the matrix interface. Under an equivalent atomic concentration, their hydrogen-trapping efficiency followed the order of NbC > TiC > VC [22]. However, the modification potential of traditional microalloy carbide systems became limited, which could not fully satisfy the increasing demand for the anti-hydrogen performance of advanced ultra-low-carbon HSLA marine steels. Accordingly, research attention gradually shifted toward nanoscale Cu-rich precipitates and their hydrogen-trapping mechanism in Cu-bearing ultra-low-carbon high-strength steels.

Previous studies verified that nanoscale Cu-rich precipitates could serve as efficient and stable hydrogen traps to improve the HE susceptibility of high-strength steels. Komazaki et al. [22] investigated the hydrogen-affected mechanical degradation of Cu-rich ultra-low-carbon steels and confirmed that the precipitate morphology significantly determined the hydrogen-induced strength deterioration. Large-sized ε-Cu particles exhibited a stronger hydrogen-trapping ability than fine Cu clusters and 9R twin-structured Cu, effectively alleviating the hydrogen-induced mechanical attenuation. Lin et al. [23] reported that Cu precipitation effectively increased the hydrogen retention time and suppressed hydrogen diffusion in tempered martensitic steel, thereby improving the HE susceptibility during slow-strain-rate tensile tests. Shi et al. [24] further demonstrated that nanoscale Cu-rich precipitates enhanced the hydrogen-induced cracking resistance through effective hydrogen immobilization rather than sacrificing ductility, breaking the traditional cognition of the ductility–HE susceptibility correlation. Subsequent comparative research conducted by Lin et al. [25] established the quantitative sequence of the hydrogen-trapping capacity at room temperature—co-precipitated ε-Cu and TiC > single TiC > grain boundary > single ε-Cu > dislocation—further confirming the superior hydrogen-trapping performance of Cu-rich nano-precipitates.

Although the hydrogen-trapping advantage of Cu-rich precipitates has been widely recognized, the dynamic evolution of nanoscale Cu-rich precipitates with different tempering durations and its quantitative correlation with HE performance in ultra-low-carbon hull structural steels remained unclear. The coupled effect of the precipitate size, number density, and dislocation configuration on hydrogen diffusion and HE sensitivity still lacked systematic elucidation, which hindered the precise microstructure optimization and engineering application of high-performance Cu-bearing marine steels. Against this background, a novel ultra-low-carbon Cu-bearing marine structural steel was designed based on the chemical composition of HSLA-100 steel in this work. The microstructure evolution, precipitate characteristics, mechanical performance, and HE susceptibility under different tempering times were systematically explored. The microstructural features and nanoscale Cu precipitate distribution were characterized via optical microscopy (OM) and transmission electron microscopy (TEM). Slow-strain-rate tensile tests (SSRTs) combined with electrochemical hydrogen charging were performed to evaluate the hydrogen embrittlement resistance. This study aimed to reveal the intrinsic relationship between the tempering-regulated Cu precipitation behavior and hydrogen embrittlement performance, providing a fundamental theoretical basis for the microstructure design and performance optimization of high-strength, tough, and hydrogen-embrittlement-resistant marine structural steels.

## 2. Materials and Methods

In the present work, a novel ultra-low-carbon hull structural steel was developed, referring to the mature composition design and manufacturing route of HSLA-100 marine steel, balancing practical service performance and production cost. The measured chemical composition of the fabricated steel (wt.%) is listed as follows: 0.02% carbon (C), 1.52% copper (Cu), 1.54% nickel (Ni), 1.0% manganese (Mn), 0.58% chromium (Cr), 0.33% silicon (Si), 0.29% molybdenum (Mo), 0.032% vanadium (V), and 0.05% aluminum (Al).

**Figure 1:**
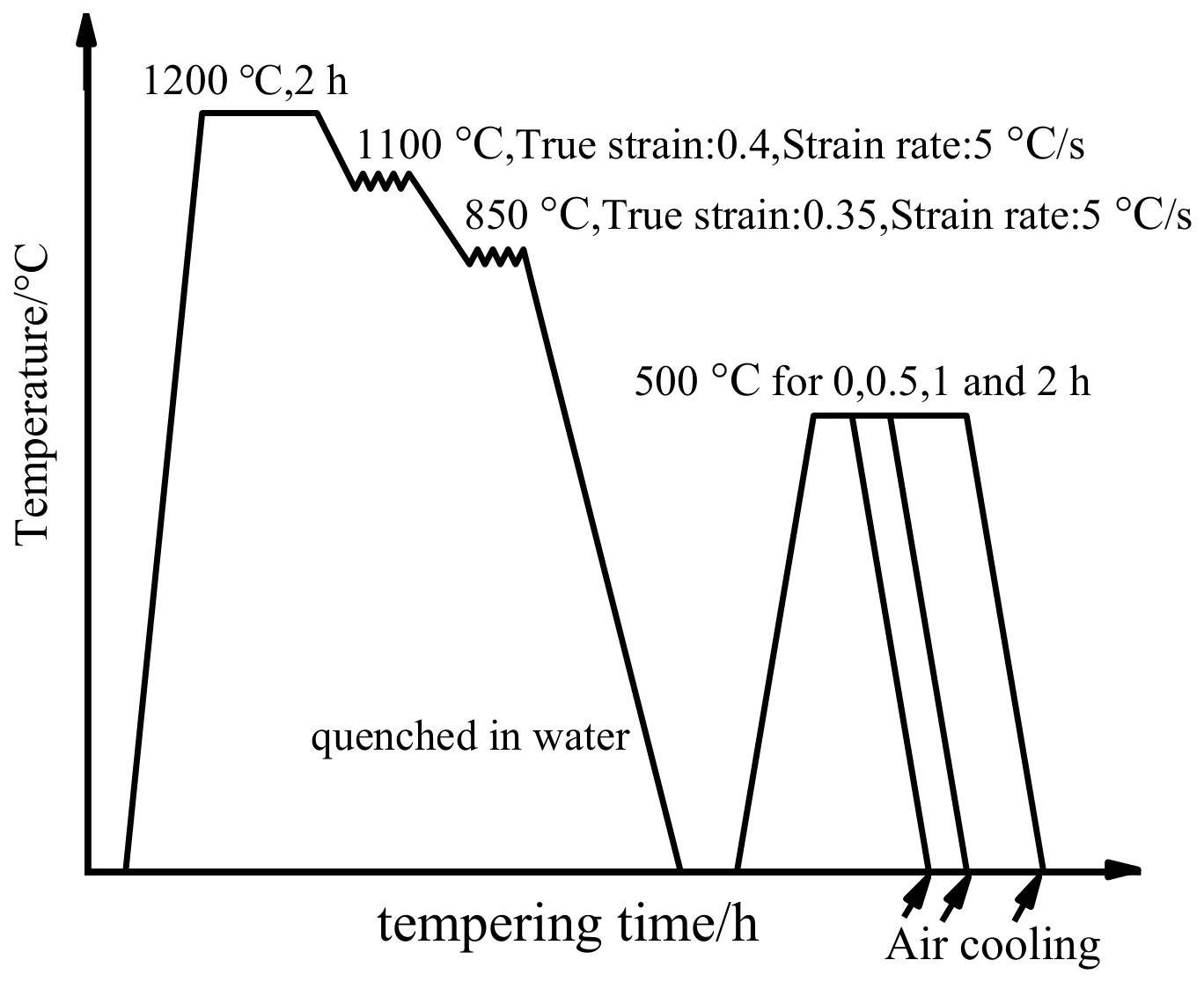
Heat treatment scheme with different tempering times.

The overall heat treatment and tempering process adopted in this experiment is schematically displayed in Figure 1. The raw steel ingot was first forged into an 80 mm-thick slab, followed by homogenization treatment at 1200 °C for 2 h to eliminate component segregation. A two-stage hot rolling procedure was implemented with an initial rolling temperature of 1100 °C, and the slab was finally processed into a finished plate with a thickness of 10 mm and a width of 100 mm. Multi-pass rolling was applied throughout the forming process to provide sufficient deformation energy for the subsequent uniform precipitation of Cu-rich nanoparticles. The start rolling temperature was set at 850 °C, and the finish rolling temperature was strictly controlled at 800 °C. Thereafter, the hot-rolled plates underwent isothermal tempering at 500 °C for four different holding durations (0 h, 0.5 h, 1 h, and 2 h), before being quenched to room temperature.

Microstructural characteristics of the tempered steel specimens were systematically characterized via optical microscopy (OM) and transmission electron microscopy (TEM, Tecnai G2 F20, FEI Company, Hillsboro, OR, USA). For OM observation, specimen surfaces were mechanically ground step-by-step using 80–1500 mesh SiC abrasive papers and polished with 2.5 μm diamond polishing suspension, followed by etching in a 2% nital solution to reveal the microstructure. For TEM specimen preparation, the bulk samples were thinned mechanically to a residual thickness of 40–50 μm, and standard 3 mm-diameter discs were punched out. Electrochemical double-jet thinning was carried out on a Tenu Pol-5 instrument (Struers ApS, Ballerup, Denmark) using a 10% nitric acid solution in anhydrous ethanol to prepare ultrathin regions for high-resolution TEM testing.

The Vickers hardness of experimental steel was measured under an indentation load of 100 g with a dwell time of 15 s. Ten repeated tests were conducted for each group of samples, and the average value was taken as the final hardness result to guarantee data reliability. Cylindrical tensile samples with a diameter of 5 mm and a gauge length of 25 mm were machined along the rolling direction of the steel plates. Quasi-static uniaxial tensile tests were performed at room temperature on a SCHENCK-100 KN electro-hydraulic servo testing machine (Schenck RoTec GmbH, Darmstadt, Germany) with a constant displacement rate of 1 mm/min, complying with the Chinese standard GB/T 228.1 [26].

Electrochemical hydrogen charging pretreatment was performed to evaluate the hydrogen embrittlement resistance of the experimental steel. For hydrogen infiltration test, tensile specimens 74 mm in length and 5 mm in diameter were used. The electrolyte solution was prepared using 0.5 mol/L dilute sulfuric acid, and 3 g/L thiourea was added as a catalytic agent to promote hydrogen atom penetration into the steel matrix. All samples were charged at a constant current density of 25 mA/cm^2^ for 75 min to obtain a stable hydrogen concentration inside the material.

## 3. Results and Discussion

### 3.1. Microstructure Analysis

**Figure 2:**
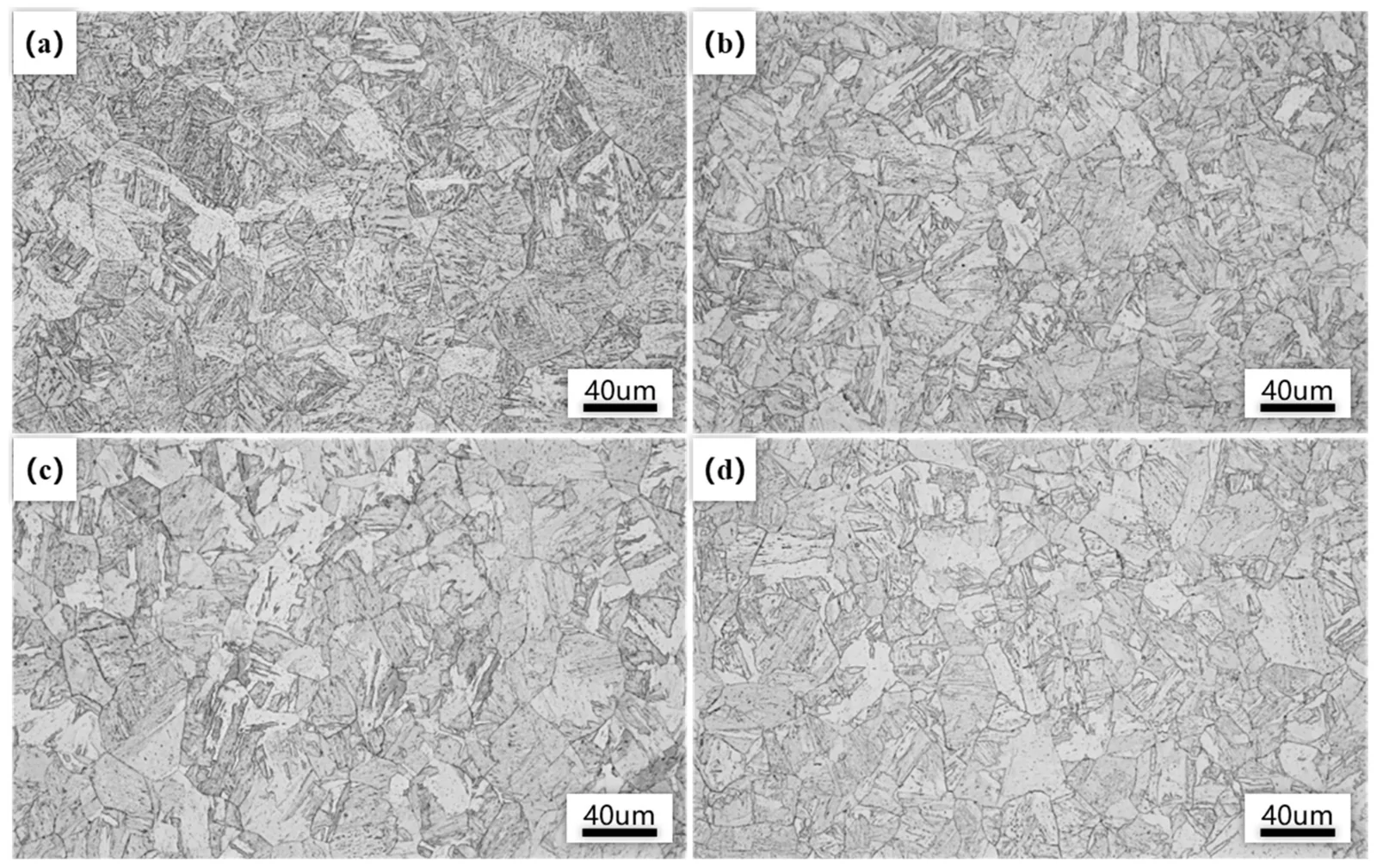
Optical microstructure of experimental steel with different tempering times: (**a**) 0 h; (**b**) 0.5 h; (**c**) 1 h; and (**d**) 2 h.

The optical micrographs of the investigated steel after different isothermal tempering times are presented in Figure 2. Microstructural observation revealed that the microstructure of the experimental steel mainly consisted of bainite and ferrite phases. As the tempering holding time increased, the original lath-shaped bainite gradually transformed into tempered bainite, accompanied by a continuous rise in the ferrite fraction. The untempered quenched microstructure (0 h, Figure 2a) consisted of typical lath bainite and ferrite, formed by water quenching from 750 °C to ambient temperature. At the early tempering stage (0.5 h, Figure 2b), the internal residual stress was fully released, and the bainite matrix underwent a typical tempering transformation. The ultra-low-carbon bainite gradually decomposed, triggering carbon redistribution and the precipitation of fine carbide particles. Such a microstructural evolution moderately reduced the hardness and improved the toughness of the steel. Moreover, the fine Cu-containing precipitates experienced a slight coarsening during tempering, which further enhanced the matrix strength of the experimental steel.

When the tempering time was extended to 1 h (Figure 2c), carbides embedded within the bainite matrix continuously coarsened and redistributed, imposing a remarkable effect on the strength and hardness of the material. Meanwhile, the partial retained austenite underwent a phase transformation to form additional ferrite and carbides, further improving the microstructural homogeneity of the steel. At the late tempering stage of 2 h (Figure 2d), the microstructure achieved a relatively stable thermodynamic state. The coarsening of carbides and the distribution of various precipitates reached a dynamic equilibrium, corresponding to a relatively low and stable hardness value. The precise control of tempering parameters can effectively tailor the microstructural features and optimize the comprehensive mechanical properties of the ultra-low-carbon Cu-bearing steel.

**Figure 3:**
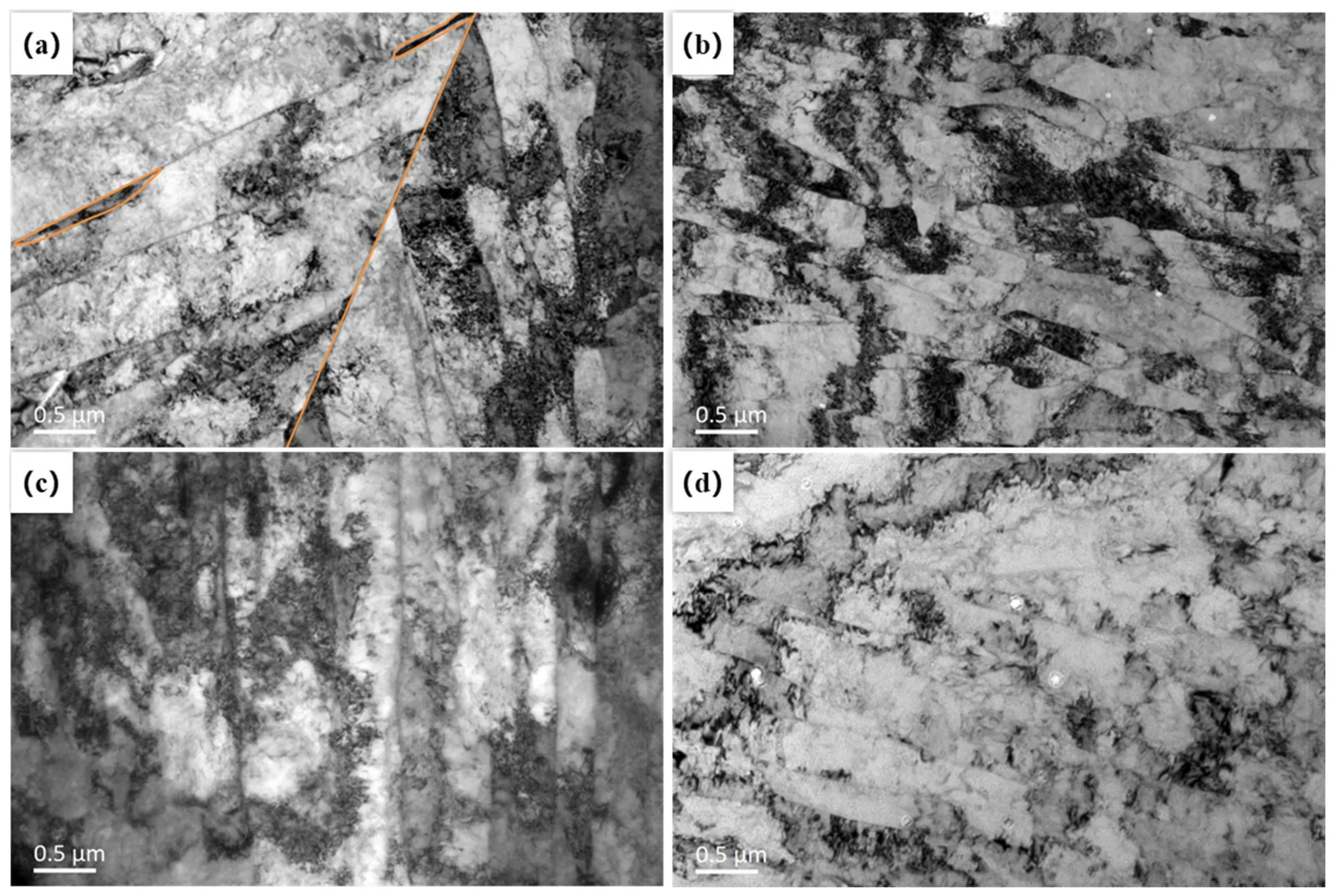
TEM morphology of experimental steel with different tempering times: (**a**) 0 h; (**b**) 0.5 h; (**c**) 1 h; and (**d**) 2 h.

TEM morphologies are displayed in Figure 3. For the untempered specimen, bainitic ferrite nucleated preferentially at the prior austenite grain boundaries, and the growth orientation of ferrite laths outlined the grain morphology of the original austenite. The bainitic ferrite exhibited an ultrafine and sharp lath structure, and the supersaturated solid-solution matrix contained a high dislocation density. Film-like or needle-shaped retained austenite was intermittently distributed along the bainite lath boundaries (Figure 3a). After tempering for 0.5 h, obvious microstructure recovery took place in the steel. The lath morphology was basically retained, whereas the lath boundaries became blurred, adjacent laths merged with one another, and the average lath width increased. The rearrangement and partial annihilation of dislocations and lattice defects effectively alleviated the lattice distortion. Numerous fine spherical precipitates were uniformly dispersed within and around the bainite laths (Figure 3b).

When the tempering time was extended to 1 h (Figure 3c), the characteristic lath features of bainite gradually faded, the lath width increased further, and the precipitates coarsened with an increased number density. After tempering for 2 h (Figure 3d), recrystallization took place in the experimental steel. The original bainitic lath microstructure disappeared completely and transformed into uniform equiaxed ferrite grains. The newly formed ferrite grains exhibited a low dislocation density, which significantly improved the microstructure stability and service performance of the material.

### 3.2. Vickers Hardness Analysis

**Figure 4:**
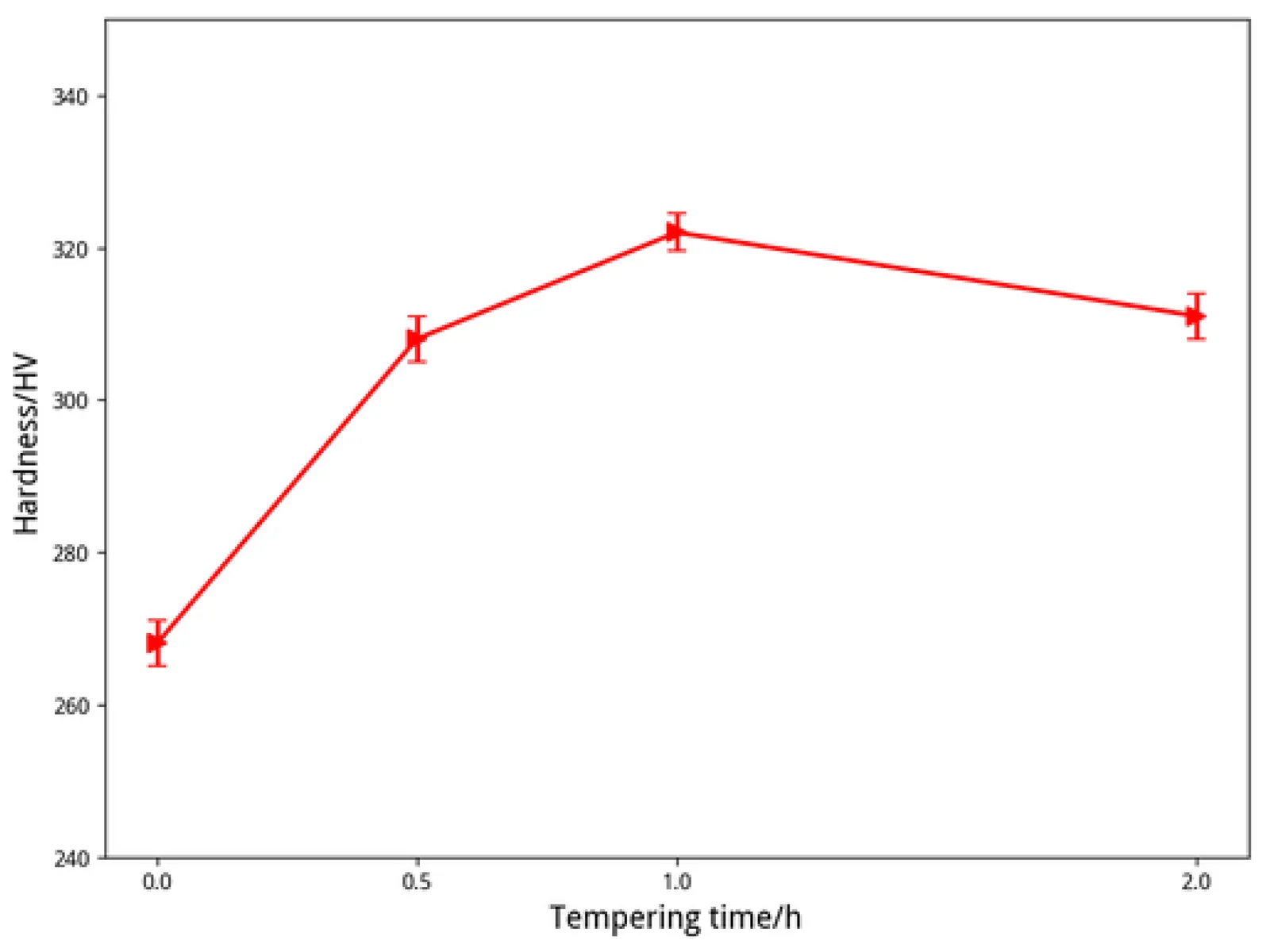
Vickers hardness of experimental steel with different tempering times.

Figure 4 presents the Vickers hardness variation with the tempering time of the experimental steel samples. The hardness exhibited a typical upward-then-downward trend with increasing tempering time, which is primarily governed by the competitive effect of precipitation strengthening and tempering softening. The untempered specimen (0 h, Figure 4) possessed the lowest hardness of 269 HV. Although the quenched bainite matrix tended to undergo stress relief, lattice distortion mitigation, and partial recrystallization during tempering, which generally reduces the hardness, the nucleation and continuous growth of Cu-rich nano-precipitates effectively offset the softening effect and promoted hardness enhancement.

After tempering for 0.5 h (Figure 4), abundant Cu atoms precipitated from the supersaturated matrix and formed fine nanoscale Cu-rich particles. The continuous increase in the precipitate size and number density increased the Vickers hardness, directly reflecting the prominent precipitation strengthening effect induced by Cu precipitation. As the tempering time was extended to 1 h (Figure 4), the growth rate of the hardness gradually slowed down, and the hardness reached a peak value of 322 HV. At this stage, the softening effect caused by microstructure recovery and recrystallization began to dominate, restraining further hardness increase. When the tempering duration was prolonged to 2 h (Figure 4), the hardness slightly decreased to 310 HV. Excessive tempering facilitated the aggregation and coarsening of tiny Cu-rich nano-particles, which reduced the quantity of effective strengthening precipitates and weakened the precipitation strengthening efficiency, thereby resulting in a moderate decline in macroscopic hardness.

### 3.3. Size and Distribution Evolution of Precipitated Particles

**Figure 5:**
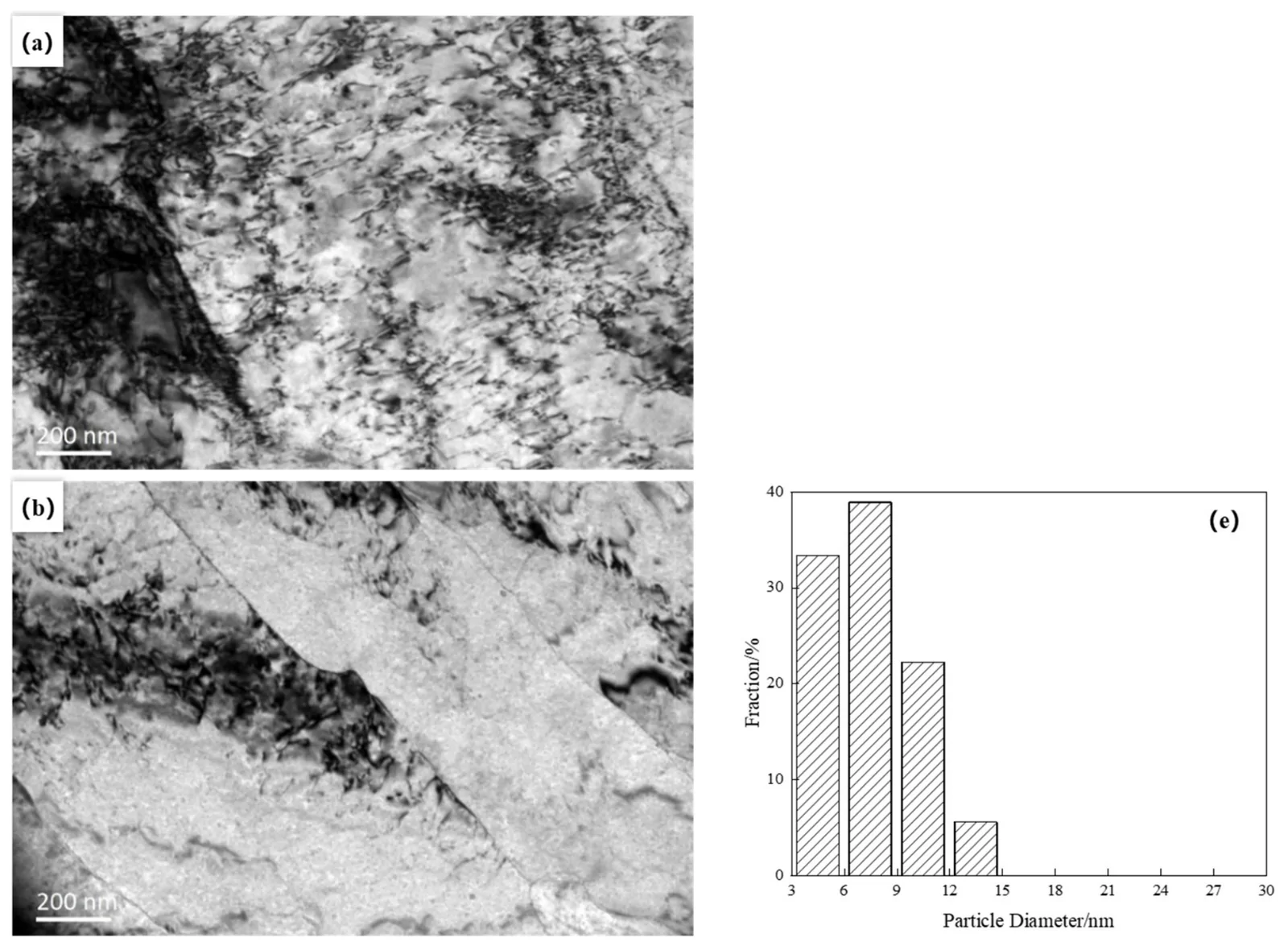

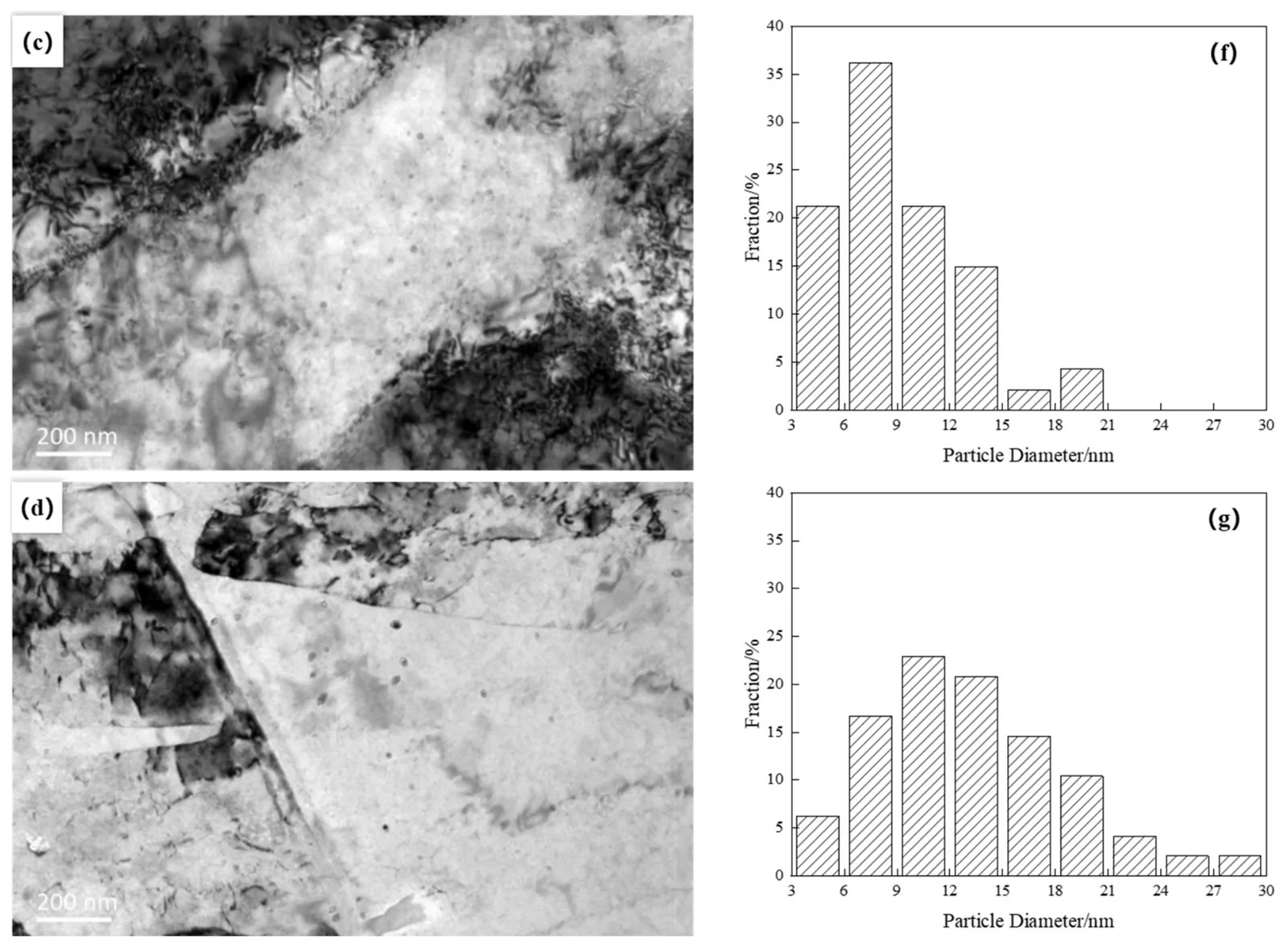
TEM micrographs and corresponding size-distribution histograms of precipitated particles in experimental steel with different tempering times: (**a**) 0 h; (**b**) 0.5 h; (**c**) 1 h; and (**d**) 2 h. Sub-figures (**e**–**g**) show particle-size histograms for 0.5 h-, 1 h-, and 2 h-tempered specimens, respectively.

Figure 5 illustrates the size distribution and morphological characteristics of precipitated particles in the experimental steel after different tempering times. No Cu-rich nano-precipitates were detected in the as-rolled specimen. This phenomenon can be attributed to abundant lattice defects, such as dislocations and grain boundaries, introduced by the severe plastic deformation during rolling. These defects acted as solid-solution sites for Cu atoms and increased the solid solubility of Cu in the steel matrix [22]. In addition, the nucleation and aggregation of precipitates require sufficient atomic migration time. The rapid water-quenching process restricted the diffusion and accumulation of Cu atoms, thereby inhibiting the formation of observable Cu-rich precipitates in the as-rolled state.

Similarly, the untempered specimen (0 h, Figure 5a) exhibited negligible Cu-rich precipitation, while a high density of tangled dislocations was widely distributed throughout the matrix. After short-term tempering for 0.5 h (Figure 5b,e), numerous fine and uniformly dispersed Cu-rich nanoparticles formed within and at the boundaries of bainite laths. The particle size ranged mainly from 3 nm to 9 nm, with an average diameter of 7.2 nm and a number density of 1.19 × 10^20^ m^−3^, indicating that rapid Cu precipitation nucleation occurred at the initial tempering stage.

As the tempering time was increased to 1 h (Figure 5c,f), the precipitates grew continuously with a size distribution of 3–12 nm, an average particle size of 10.2 nm, and an elevated number density of 3.3 × 10^20^ m^−3^. The simultaneous increase in precipitate size and number density demonstrated the significant enhancement in precipitation strengthening. When the tempering duration was further prolonged to 2 h (Figure 5d,g), the Cu-rich particles continued to coarsen, with a size range of 6–18 nm and an average size of 13.2 nm, while the number density increased only slightly to 3.37 × 10^20^ m^−3^. The stable number density indicated that precipitate growth reached a dynamic saturation state, and further extension of tempering time exerted a limited influence on precipitate quantity.

The reduction in hardness after tempering for 2 h was mainly associated with the recrystallization of the bainite matrix. The newly formed equiaxed ferrite grains possessed a lower dislocation density and weaker lattice distortion, which induced an obvious softening effect and offset the strengthening contribution from coarsened Cu-rich precipitates.

In the present study, the average size of Cu-rich precipitates increases from 7.2 nm to 13.2 nm with a prolonged tempering time, which falls within a comparable size range reported by Komazaki et al. and Lin et al. for Cu-bearing steels. Previous studies have indicated that fine Cu-rich clusters below 10 nm possess a weak hydrogen-trapping capacity, while the hydrogen-trapping efficiency is remarkably enhanced once Cu-rich precipitates grow above 10 nm to form the ε-Cu phase. This is consistent with our experimental observations: severe hydrogen-embrittlement damage occurs for the 0.5 h-tempered sample with an average precipitate size of 7.2 nm, whereas HE susceptibility is improved for the 1 h-tempered sample whose precipitates exceed 10 nm in average dimension.

Most prior investigations mainly focused on the hydrogen-trapping behavior of Cu-rich precipitates themselves. By contrast, this work further reveals that tempering simultaneously modulates both precipitate characteristics and dislocation configurations. A high dislocation density does not unconditionally mitigate hydrogen embrittlement; the final performance depends strongly on whether the dislocations are dominated by tangled-sessile structures or mobile ones.

### 3.4. Mechanical Property Analysis

**Figure 6:**
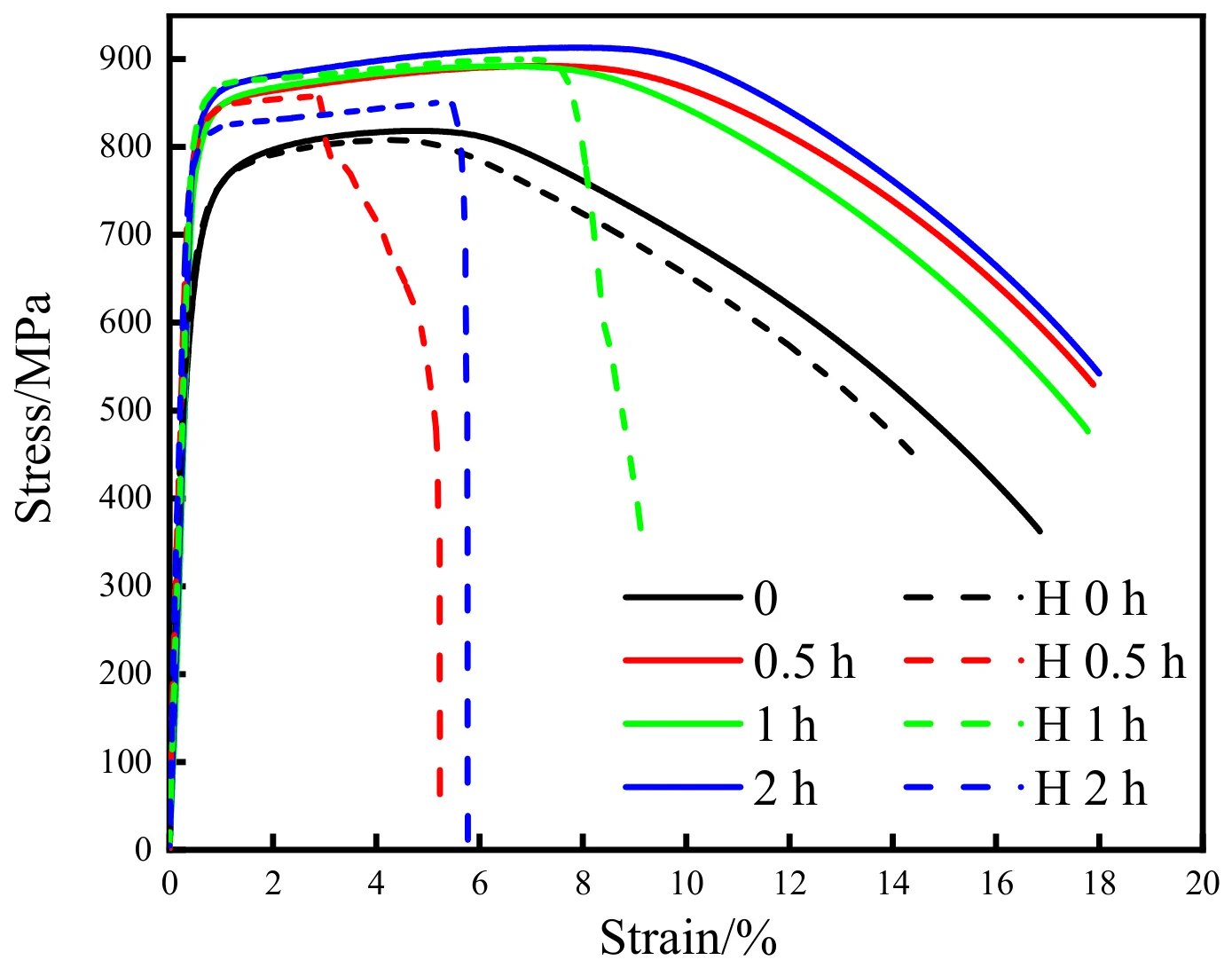
Comparison of tensile properties of experimental steel before (solid line) and after (dashed line) hydrogen charging.

The tensile properties of the experimental steel before and after hydrogen charging under different tempering conditions are presented in Figure 6. For hydrogen-free specimens, the tempered samples exhibited a significantly higher strength and slightly improved elongation compared with the as-rolled sample. During tempering, supersaturated Cu atoms gradually precipitated from the matrix and formed fine Cu-enriched domains or second-phase particles. These nanoscale precipitates effectively hindered the dislocation motion, thereby improving both the yield strength and tensile strength. In addition, tempering homogenized the microstructural distribution, relieved local stress concentration, and promoted dislocation rearrangement and annihilation, which reduced the residual internal stress and, correspondingly, enhanced the tensile ductility [8].

For hydrogen-charged specimens, the as-rolled sample retained the highest post-charging elongation, followed by the sample tempered for 1 h, while the specimens tempered for 0.5 h and 2 h showed the poorest ductility. Combined with the microstructural characteristics shown in Figure 5a, no distinct Cu-rich precipitates were observed in the as-rolled steel, whereas a large number of high-density tangled dislocations existed in the matrix. These tangled dislocations acted as irreversible hydrogen traps, provided abundant hydrogen trapping sites, and redistributed internal hydrogen concentration. Such microstructural features modified the critical conditions for hydrogen-induced cracking and effectively suppressed hydrogen embrittlement failure [23].

Compared with hydrogen-free groups, the as-rolled specimen exhibited the minimum mechanical degradation after hydrogen charging, with an elongation loss rate of only 14.33% and a reduction in the area loss rate of 9.2% (Table 1). During tempering, thermal activation provided sufficient energy for atomic rearrangement, which promoted the migration and annihilation of dislocations. A considerable portion of tangled dislocations generated by the rolling deformation were untied through dislocation slip and climb, resulting in a simplified and regular dislocation configuration and an overall decrease in the dislocation density.

The as-rolled sample exhibited the minimum plastic loss after hydrogen charging. This behavior originates from large quantities of high-density tangled, sessile dislocations introduced by hot rolling deformation. Such tangled dislocation forests can act as relatively stable hydrogen traps, pinning the diffusible hydrogen and redistributing the hydrogen concentration within the matrix to alleviate local hydrogen enrichment.

It should be noted that dislocations exert dual effects on hydrogen embrittlement, and a high dislocation density alone cannot guarantee an improved HE susceptibility. During short-time tempering (0.5 h), recovery untangles part of the dislocation networks and generates abundant mobile dislocations. Mobile dislocations are capable of transporting hydrogen atoms toward grain boundaries and crack tips during plastic deformation, which promotes local hydrogen accumulation and aggravates the hydrogen-induced damage. Accordingly, HE susceptibility is governed by the dislocation configuration rather than simple dislocation density: microstructures dominated by tangled-sessile dislocations can serve as hydrogen traps, while structures with abundant mobile dislocations tend to accelerate hydrogen-induced degradation.

With a prolonged tempering time, the dislocation density continuously decreased, while the size and number density of Cu-rich precipitates gradually increased. The specimen tempered for 0.5 h presented a severe ductility loss after hydrogen charging, with an elongation loss rate of 70.85% and a reduction in the area loss rate of 86.4%. At this stage, the number density of Cu-rich precipitates was relatively low, and the average particle size was less than 10 nm. According to a previous microstructural analysis, Cu-rich particles smaller than 10 nm generally possess a BCC Fe–Cu structure [8]. In comparison, FCC Cu phases with larger interstitial spaces and higher structural symmetry exhibit a superior hydrogen trapping capacity. Meanwhile, short-time tempering retained a large number of mobile dislocations, which have been widely reported to act as harmful reversible hydrogen traps and accelerate hydrogen-induced deterioration. The combined effects of insufficient high-efficiency Cu-rich traps and excessive mobile dislocations led to severe embrittlement in the 0.5 h-tempered sample.

In contrast, the specimen tempered for 1 h exhibited relatively low mechanical degradation after hydrogen charging, with an elongation loss rate of 48.63% and a reduction in the area loss rate of 52.8%. The extended tempering promoted the continuous growth and uniform distribution of Cu-rich precipitates, whose average size exceeded 10 nm with a significantly increased number density. After 1 h tempering, adequately grown Cu-rich precipitates with a high number density are inferred to act as stable hydrogen-trapping sites according to previous literature, which can immobilize diffusible hydrogen atoms and suppress long-range hydrogen migration. The restriction of the hydrogen diffusion reduced the hydrogen accumulation at critical defect sites such as crack tips, thereby improving the hydrogen embrittlement resistance. Moreover, the uniformly distributed Cu-rich precipitates adjusted the local stress field and changed the hydrogen migration path, further inhibiting the hydrogen concentration in brittle-prone regions. Synchronously, the density of harmful mobile dislocations is substantially reduced in the matrix. The combined contributions of precipitates and dislocations collectively improve the HE susceptibility of the experimental steel.

Nevertheless, further prolonging the tempering duration to 2 h aggravated the hydrogen-induced ductility loss again, with the elongation loss rate rising to 67.98% and the reduction in the area loss rate reaching 85.6%. Although the continuously coarsened Cu-rich precipitates and further decreased dislocation density were beneficial for hydrogen trapping, the tempering temperature of 500 °C lies within the typical temperature range for the reversible temper brittleness of low-alloy steels. Combining the severe plasticity deterioration after hydrogen charging and prominent intergranular fracture features of the 2 h-tempered specimen, it is inferred that long-term isothermal holding triggers reversible temper-brittleness-related degradation. During prolonged tempering, bainite decomposes, alloy carbides such as CrC, MoC, and VC form, and the retained austenite transforms into cementite. Impurity elements including P, Sn, and Sb tend to segregate toward grain boundaries, which weakens the grain-boundary cohesion. Hydrogen atoms preferentially accumulate at these weakened grain boundaries and phase boundaries and accelerate the brittle fracture.

The hydrogen-charged tensile results demonstrated that the as-rolled steel possessed the lowest hydrogen-induced mechanical loss due to the protective effect of high-density tangled dislocations. Severe embrittlement occurred in the 0.5 h- and 2 h-tempered specimens; insufficient high-efficiency Cu traps and excessive mobile dislocations dominated the embrittlement of the 0.5 h sample, whereas reversible temper brittleness accounted for the deterioration of the 2 h sample. The 1 h-tempered steel achieved the optimal hydrogen embrittlement resistance, which is attributed to the moderate precipitate size, high number density of Cu-rich nano-phases, and significantly reduced mobile dislocation density.

### 3.5. Fracture Morphology Analysis

**Figure 7:**
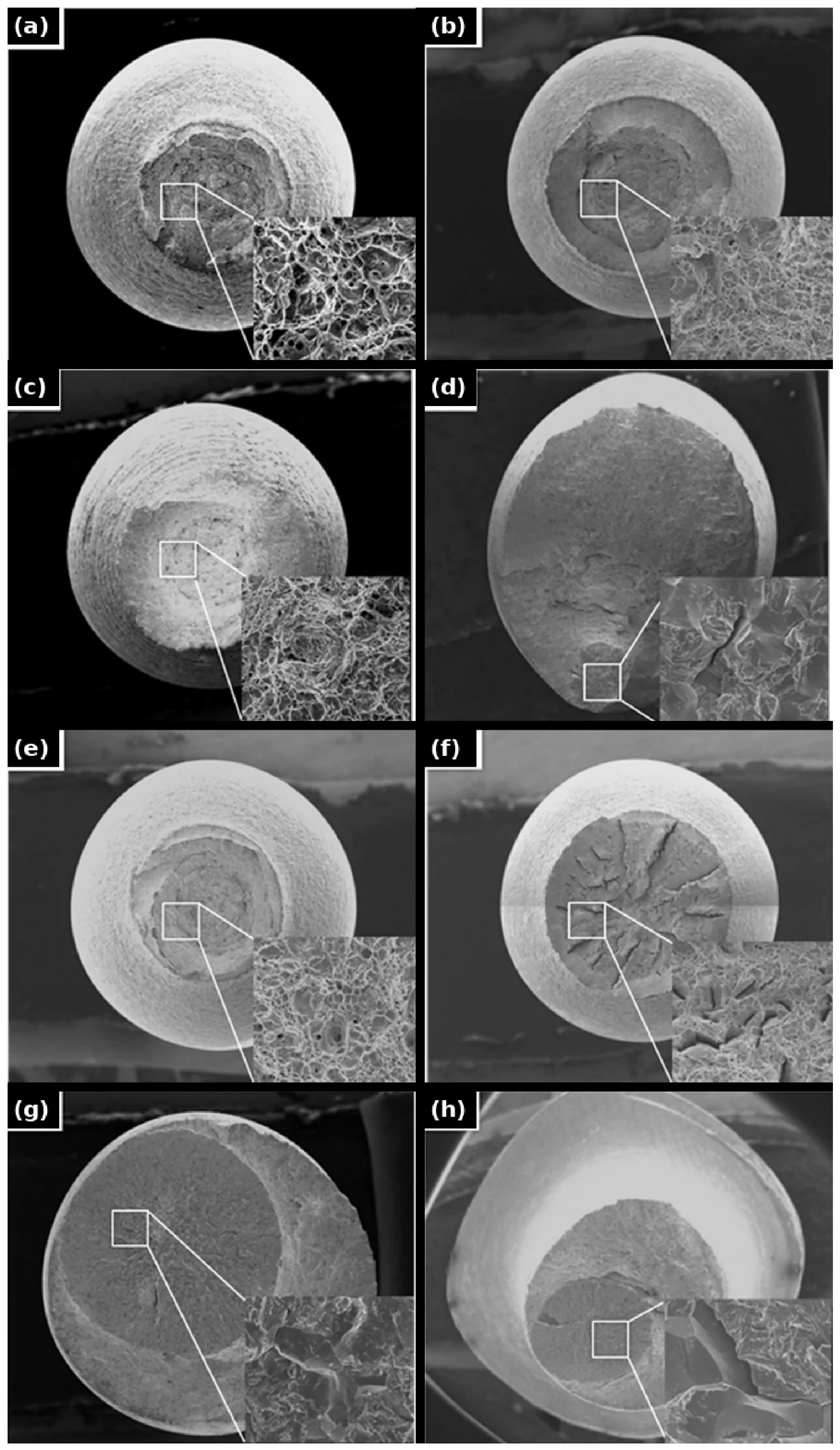
Tensile fracture morphologies of experimental steel under different tempering times (**left** column: specimens without hydrogen charging; **right** column: specimens after electrochemical hydrogen charging): (**a**,**b**) 0 h; (**c**,**d**) 0.5 h; (**e**,**f**) 1 h; and (**g**,**h**) 2 h. Insets show magnified local fracture features.

Figure 7 presents the tensile fracture morphologies of the tested steel under different tempering durations. In metallic materials, hydrogen atoms tended to accumulate around dislocations and form Cottrell atmospheres [27,28]. Such aggregation reduced the elastic strain energy of dislocations and increased the susceptibility to dislocation slip, thereby inhibiting the plastic deformation of the matrix and inducing hydrogen embrittlement. During the deformation at low strain rates, hydrogen atoms migrated along with moving dislocations and redistributed the internal hydrogen concentration. Since dislocations preferentially accumulated near grain boundaries, localized hydrogen enrichment occurred at grain boundary regions. The coupling effect of the elevated hydrogen concentration and internal stress ultimately triggered intergranular cracking and exacerbated material embrittlement [29,30,31].

All hydrogen-free specimens exhibited typical ductile fracture characteristics. The tempered specimens possessed a larger number of dimples on the fracture surfaces compared with the as-rolled specimens, indicating that tempering optimized the matrix microstructure and improved the plastic performance. Nevertheless, numerous fine and disorderly oriented microcracks were observed on the fracture surface of the specimen tempered for 2 h, which was closely associated with the occurrence of reversible temper brittleness.

After hydrogen charging, the fracture mechanism of the tested steel changed significantly, owing to the hydrogen penetration and aggregation, as well as microstructural differences. The hydrogen-charged as-rolled specimens still retained distinct dimple characteristics with partial river-like patterns on the fracture surface, leading to a slight deterioration in tensile elongation and a relatively low degree of embrittlement. This phenomenon was attributed to the dual effects of hydrogen traps in the material. Precipitates in the tested steel acted as effective hydrogen traps. During deformation, mobile dislocations deliver diffusible hydrogen in the matrix to trap sites. This immobilizes the free hydrogen and homogenizes the hydrogen distribution, relieving the local hydrogen accumulation and hydrogen embrittlement damage. However, the stress concentration around hydrogen traps nucleates sparse microcracks and slightly degrades the plasticity of steel [32].

In sharp contrast, the hydrogen-charged tempered specimens exhibited nearly vanished dimples and prominent intergranular fracture features, indicating severe hydrogen-induced embrittlement. Furthermore, the preferential accumulation of dislocations at grain boundaries further aggravated the hydrogen enrichment in these regions. The synergistic effect of a high hydrogen concentration and internal residual stress promoted the initiation and rapid propagation of intergranular cracks, which substantially impaired the plastic deformation capacity of the matrix and eventually resulted in typical hydrogen-induced brittle fracture. Grain refinement improved the HE susceptibility of steel to the hydrogen-induced delayed fracture by increasing the grain boundary density, homogenizing the hydrogen distribution, and suppressing the localized hydrogen aggregation [33]. By contrast, inappropriate tempering processes weakened this protective mechanism and significantly increased the hydrogen embrittlement susceptibility of the material.

## 4. Conclusions

In this work, the correlations between the tempering duration, Cu-rich nano-precipitate characteristics, and hydrogen embrittlement performance of ultra-low-carbon Cu-bearing marine steel were investigated. The primary conclusions are summarized as follows:

(1) With the extension of the tempering time, the initial lath bainite microstructure of the experimental steel gradually transformed into tempered bainite and equiaxed ferrite. Benefiting from the prominent precipitation strengthening of nanoscale Cu-rich precipitates, the tempered specimens exhibited a significantly improved yield strength and tensile strength, as well as a slight enhancement in ductility, compared with the as-rolled counterpart.

(2) Electrochemical hydrogen charging tests were performed to clarify the regulation mechanism of the Cu-rich precipitate size and number density on the hydrogen embrittlement behavior under different tempering conditions. As the tempering duration increased, the microstructure underwent continuous evolution from quenched bainite to a stabilized tempered microstructure. The average size of Cu-rich precipitates increased from 7.2 nm after 0.5 h of tempering to 13.2 nm after 2 h of tempering, while the precipitate number density rose rapidly at the initial tempering stage and finally reached a saturated stable state.

(3) Hydrogen-charged tensile tests demonstrate that nanoscale Cu-rich precipitates provide precipitation-strengthening effects in the investigated ultra-low-carbon steel. Combined with the microstructural observations and previously reported literature, it is inferred that Cu-rich precipitates with a proper size and high number density, together with favorable tangled-dislocation configurations, can suppress the diffusion and local aggregation of diffusible hydrogen. The synergistic optimization between the precipitate microstructure and dislocation morphology contributes to an improved hydrogen-embrittlement resistance. Direct hydrogen-trapping measurements are lacking in this study, and the relevant mechanism needs further validation via dedicated experiments in future work.

## Figures and Tables

**Table 1:** The loss rate of fracture extension and section shrinkage of experimental steel before and after hydrogen charging.

Tempering Time/h	Elongation Loss Rate/%	Section Shrinkage Loss Rate/%
0	14.33	9.2
0.5	70.85	86.4
1	48.63	52.8
2	67.98	85.6

## Data Availability

The original contributions presented in this study are included in the article. Further inquiries can be directed to the corresponding authors.
